# The role of γδ T cells in flavivirus infections: Insights into immune defense and therapeutic opportunities

**DOI:** 10.1371/journal.pntd.0012972

**Published:** 2025-04-17

**Authors:** Qi Li, Meng Zhang, Bridget Kim, Samuel Soriano, Hridesh Mishra, Qiuyue Wang, Kevin C. Kain, Ran Wang

**Affiliations:** 1 Laboratory of Infection and Virology, Beijing Pediatric Research Institute, Beijing Children’s Hospital, Capital Medical University, National Center for Children’s Health, Beijing, China; 2 Research Unit of Critical Infection in Children, 2019RU016, Chinese Academy of Medical Sciences, Beijing, China; 3 Department of Endocrinology, The First Affiliated Hospital of China Medical University, Shenyang, Liaoning, China; 4 Department of Pediatric Rehabilitation, Beijing Boai Hospital, School of Rehabilitation Medicine, Capital Medical University, China Rehabilitation Research Center, Beijing, China; 5 Sandra A. Rotman (SAR) Laboratories, Sandra Rotman Centre for Global Health, University Health Network-Toronto General Hospital, Toronto, Ontario, Canada; 6 Tropical Disease Unit, Division of Infectious Diseases, Department of Medicine, University of Toronto, Toronto, Ontario, Canada; 7 Department of Experimental Therapeutics, University Health Network-Toronto General Hospital, Toronto, Ontario, Canada; 8 Faculty of Medicine, University of Toronto, Toronto, Ontario, Canada; La Jolla Institute for Immunology, UNITED STATES OF AMERICA

## Abstract

γδ T cells are a unique subset of unconventional T cells and an important component of the innate immune system. Unlike conventional αβ T cells, γδ T cells can respond rapidly during the early stages of infection, and their antigen recognition is not restricted by MHC molecules. These distinctive features underscore the important role of γδ T cells in viral clearance and infection control. Therefore, γδ T cell-based immunotherapies have been extensively explored for the treatment of a variety of diseases, including viral infections and cancers. Several therapeutic strategies based on γδ T cells have advanced to clinical trials, demonstrating promising safety and efficacy. Currently, there are no effective treatments for flavivirus infections, which are typically characterized by acute onset. Research has shown that γδ T cells can rapidly expand during the early phases of flavivirus infections and effectively suppress viral replication, making them an attractive target for the development of novel therapies for flavivirus infections. This review aims to highlight the immunological roles of γδ T cells in flavivirus infections and to explore the potential of γδ T cell-based therapeutic strategies for the prevention and treatment of these infections.

## Background

Mosquito-borne flaviviruses belong to the genus *Orthoflavivirus* of the family *Flaviviridae*, which are enveloped, single-stranded positive-sense RNA viruses. They are primarily transmitted by various mosquito species such as *Aedes* and *Culex*. Key members of this genus include Dengue virus (DENV), Zika virus (ZIKV), West Nile virus (WNV), Yellow fever virus (YFV), and Japanese encephalitis virus (JEV) [1]. Each year, these key mosquito-borne flaviviruses infect approximately 300 million people, with 100 million cases manifesting clinically. This poses a significant threat to nearly half of the world’s population and results in a substantial global disease burden [2,3]. While most infections are asymptomatic, symptomatic individuals typically present with nonspecific symptoms such as fever and rash. However, some patients may develop severe, life-threatening complications including hemorrhagic syndrome and encephalitis [4]. Due to the acute onset of flavivirus infections, adaptive immune responses are limited and the role of the innate immune response is essential to control infection.

γδ T cells, a crucial component of the innate immune system, are characterized by recognizing target cells in a non-MHC-restricted manner [5]. These cells exhibit antiviral capabilities through direct and indirect antiviral functions [6,7]. Therefore, there is a growing interest in γδ T cell-based therapeutic strategies for viral infections [7]. For example, γδ T cell specific activator amino-bisphosphonate, which has been approved for the treatment of osteoporosis and Paget’s disease, was shown to improve the outcomes in influenza virus-infected mice through selectively activating γδ T cells [8]. Moreover, γδ T cell-based immunotherapies have been applied in clinical cancer treatment, achieving efficacy and showing a promising application future [9]. Given the current lack of effective treatments for flavivirus infections and prospect of γδ T cells [10], this review summarizes the research on γδ T cells in flavivirus infections, providing a comprehensive understanding of their role and discussing the potential of γδ T cell-based therapies.

## γδ T cells

Human γδ T cells constitute approximately 1-10% of total T cells in peripheral blood, and γδ T cells are also enriched in various peripheral tissues, such as the skin and intestines, where they promote tissue homeostasis [11]. Similar to αβ T cells, γδ T cells undergo DNA rearrangement in their receptor genes (*TRD* and *TRG*). *TRD* genes include 3 specific *TRDV* genes (*V*δ*1*, *V*δ*2*, *V*δ*3*), which are most frequently utilized among the eight Vδ variants and thus are used in the classification of γδ T cell subtypes [11].

### Vδ2 T cells

Vδ2 T cells are the predominant subset in peripheral blood, comprising approximately 60%–95% of γδ T cells. During rearrangement, the Vδ2 chain almost exclusively pairs with the Vγ9 chain, resulting in Vγ9Vδ2 T cells being the most prevalent γδ T cell subset in peripheral blood [5]. As an MHC-unrestricted immune cell, γδ T cells have recently been shown to utilize a non-restricted recognition mechanism that is associated with Vγ9. Phosphoantigens (pAgs), which accumulate in infected cells, do not directly interact with Vγ9Vδ2 T cells but instead function as “molecular glues,” promoting intracellular interactions between butyrophilin subfamily 3 member A1 (BTN3A1) and BTN2A1 within the target cells. This interaction enables the extracellular domain of BTN2A1 to bind to TCR Vγ9 chain, thereby activating γδ T cells [12]. Therefore, the use of pAgs or BTN monoclonal antibodies can specifically activate Vγ9Vδ2 T cells.

Besides Vγ9Vδ2 TCR-dependent recognition pattern, Vδ2 T cells can recognize virus-infected cells through the upregulation of natural killer group 2 member D (NKG2D) ligands, such as MICA, MICB, and ULBP, on the surface of infected cells via NKG2D and induce target cell apoptosis through the Fas-Fas ligand (Fas-FasL) and TNF-related apoptosis-inducing ligand-death receptor 5 (TRAIL-DR5) death pathways [13]. Moreover, Vδ2 T cells can also express Fc gamma receptor III (CD16) and enhance their antiviral activity through antibody-dependent cellular cytotoxicity [14].

### Vδ1 T cells

Vδ1 T cells are less abundant in peripheral blood, but they are widely distributed in peripheral tissues such as intestinal epithelium, skin, liver, and spleen, contributing to tissue homeostasis [11]. In contrast to Vδ2 T cells, Vδ1 T cells do not pair with a specific Vγ chain, nor do they pair with Vγ9, and thus cannot be activated by pAgs [15]. Several MHC-like proteins (presenting lipids or metabolites), such as members of the CD1 family and the MHC class I-related molecule (MR1), have been shown to serve as ligands for Vδ1 T cells [16]. Interestingly, Vδ1 T cells can also recognize target cells through their TCR by interacting with the MHC II complex HLA-DR, thus broadening the scope of MHC-restricted recognition by Vδ1 T cells [6]. Like Vδ2 T cells, Vδ1 T cells are capable of recognizing target cells via the NKG2D pathway, while other molecules, such as NKR and DNAM-1, further enhance their cytotoxic activity [15,17]. These diverse recognition mechanisms confer enhanced immune effector functions.

### Vδ3 T cells

Vδ3 T cells are almost undetectable in the peripheral blood of healthy individuals but showed increased levels in the peripheral blood of patients with reactivated human cytomegalovirus [18]. Vδ3 T cells are predominantly located in the intestine and lamina propria [11]. They exhibit functional similarities to Vδ1 T cells, as MR1 and CD1d have also been shown to bind to Vγ8Vδ3 T cells [19,20]. However, Annexin A2, which is upregulated on tumor cells, has been found to specifically stimulate the proliferation of Vγ8Vδ3 T cells [21]. Nevertheless, research on Vδ3 T cells remains very limited.

In mice, γδ T cells are categorized into seven subgroups (Vγ1-7) based on *TRGV* genes and mouse γδ T cells lack similar Vγ9 chain, rendering them incapable of activation by pAgs [22]. Moreover, there are also differences in tissue distribution between mouse γδ T cells and human γδ T cells [23]. For example, mouse epidermal T cells consist exclusively of Vγ5^+^ dendritic epidermal T cells, whereas the human epidermis contains both αβ T cells and γδ T cells, with Vδ1 T cells present in both the epidermis and dermis [23]. Although there are differences in TCR composition and tissue specificity, mouse γδ T cells exhibit similar functional characteristics with human γδ T cells. For instance, CD1d serves as a ligand for Vδ1 T cells in both mice and humans [24]. Moreover, human and mouse epidermal γδ T cells can both produce keratinocyte growth factor and insulin-like growth factor 1, promoting wound healing [22]. Therefore, data from mouse models can provide valuable insights into understanding the role of γδ T cells in human.

## γδ T cells in flavivirus infections

Human infection with flaviviruses typically occurs through the bite of infected mosquitoes, which inject the virus into skin. The virus then spreads through the blood or lymphatic system to target organs for replication. This section will demonstrate the characteristics of γδ T cells from the process of flavivirus infections.

### The activation and amount of γδ T cells

Following a mosquito bite, the skin is the initial site of flavivirus infections where tissue-resident γδ T cells are first activated. To mimic the natural infection route via skin bites, researchers infected C57BL/6 mice with DENV using footpad injections [25]. They reported that a significant expansion of γδ T cells was observed at the initial infection site (footpad) and in draining lymph nodes (DLNs) of the footpad, as early as 1 day post-infection. Moreover, γδ T cells were the most abundant T cell subset at footpad [25]. Similarly, in C57BL/6 mice intraperitoneally injected with WNV, a significant increase in γδ T cell numbers was detected in the peritoneal cavity 2 days post-infection, and γδ T cells exhibited greater proliferation compared to αβ T cells in splenocytes [26]. These findings suggest that during flavivirus infections, γδ T cells are the first T cells to respond in the early stage of infection and undergo significant expansion.

Upon entry into the blood, flaviviruses are disseminated to target organs via blood circulation. γδ T cells in peripheral blood recognize infected cells and are activated. In peripheral blood mononuclear cells (PBMCs) of patients infected with DENV, the proportion of Vδ2 T cells decreased; however, the proportion of cells expressing CD38 and HLA-DR (activation markers) remained significantly elevated, indicating that Vδ2 T cells were still in an activated state during DENV infection [27]. However, in patients with ZIKV infection, there was a significant expansion of the CD3^+^ CD4^−^ CD8^−^ (double negative, DN) T cell subset in PBMCs, which primarily consist of γδ T cells (Vδ1, Vδ2, Vδ1^−^ Vδ2^−^) and NKT cells. Flow cytometry analysis revealed that approximately 80% of DN T cells were Vδ2 T cells, a significantly higher proportion than that in control groups [28]. Moreover, the proportion of Vδ2 T cells increased markedly in the early stages of symptom onset (2–3 days) and gradually decreased with the progression of the infection. Of note, the study reported that the proportion of Vδ1^−^ Vδ2^−^ DN T cells in PBMCs of ZIKV-infected patients was 15.9%, significantly higher than the proportion of Vδ1 T cells (3.2%) [28]. In summary, the γδ T cell population in PBMCs undergoes significant alterations during the early stage of infection, indicating their potential involvement in the initial immune response to flavivirus infections.

Additionally, ZIKV infection is known to cause congenital Zika syndrome, characterized by fetal microcephaly [29]. One research had shown that following ZIKV infection in pregnant rhesus macaques, there was a significant decrease in the proportion of HLA-DR^+^ and Ki67^+^ (a proliferation marker) γδ T cells in the decidua, which is the maternal-fetal interface rich in immune cells [30]. Similarly, compared to normal pregnant macaques, the proportion of Ki67^+^ γδ T cells in PBMCs of the infected group also significantly decreased [30]. These findings suggest that ZIKV infection in pregnant macaques may suppress the activation and proliferation of γδ T cells in both the decidua and PBMCs. In addition, the immune characteristics of γδ T cells in flavivirus infections are presented in **Table 1**.

**Table 1:** Immune characteristics of γδ T cells in flavivirus infections. Human γδ T cells can primarily be divided into three main subsets: Vδ1, Vδ2, and Vδ3. γδ T _EMRA_ cells, as effector T cells, can directly kill virus-infected cells, while γδ T_EM_ cells, as central memory cells, often secrete cytokines such as IFN-γ to exert indirect antiviral effects.

Viruses	Samples	Proportion	Phenotype	References
DENV	PBMCs from patients	Vδ2 T↓	γδ T_EMRA_↑ γδ T_EM_↓	[27]
	Cells in footpad and draining lymph node from mice	γδ T↑		[25]
ZIKV	PBMCs from patients	Vδ2 T↑, Vδ1^−^ Vδ 2^−^ T↑	γδ T_EMRA_↑, γδ T_EM_↓	[28]
	PBMCs and placenta from pregnant monkeys	γδ T↓	In PBMCs, γδ T_EMRA_↑, γδ T_EM_↓ In placenta, γδ T_EMRA_↓	[29]
WNV	Spleen and peritoneal cells from mice	γδ T↑		[26]

DENV, dengue virus; ZIKV, Zika virus; WNV, West Nile virus; PBMCs: peripheral blood mononuclear cells; IFN-γ, interferon-γ.

### The cellular phenotype of γδ T cells

Similar to αβ T cells, γδ T cells can also be categorized into four subsets based on cellular phenotypes: naïve (γδ T_N_), central memory (γδ T_CM_), effector memory (γδ T_EM_), and terminally differentiated effector cells (γδ T_EMRA_). Indeed, the differentiation phenotype of γδ T cells is closely associated with their functional phenotype. γδ T_EM_ and γδ T_EMRA_, as major effector cells, exhibit distinct functions: γδ T_EM_ typically secretes Th1-type cytokines such as IFN-γ and TNF-α, while γδ T_EMRA_ primarily engages in cytotoxic activity against target cells through the secretion of perforin, granzyme B, and NK cell receptors-mediated mechanisms [14].

In PBMCs of patients infected with DENV and ZIKV, there was a notable increase in the proportion of granzyme B^+^ γδ T cells (corresponding to γδ T_EMRA_ cells), while the proportion of IFN-γ^+^ γδ T (γδ T_EM_) cells showed a declining trend [27,28]. Of note, it can be observed that the expression levels of TIM-3 were significantly negatively correlated with the proportion of IFN-γ^+^ γδ T cells in dengue patients, suggesting that TIM-3 may negatively regulate the secretion of IFN-γ [27]. Similarly, comparable changes were observed in PBMCs of pregnant monkeys infected with ZIKV, with an increase in the proportion of γδ T_EMRA_ cells and a decrease in the proportion of γδ T_EM_ cells [30]. However, in the decidua of pregnant macaques infected with ZIKV, there was a significant decrease in the proportion of granzyme B^+^ γδ T (γδ T_EMRA_) cells compared to normal pregnant monkeys, indicating an impairment in the protective function of γδ T cells [30]. Taken together, enhancing the function of γδ T cells holds potential to benefit patients by augmenting their immune response.

In addition to the functions mentioned above, γδ T cells can also secrete IL-17A. In C57BL/6 mice infected with DENV, γδ T cells underwent significant expansion by day 4 post-infection and were the major producers of IL-17A in the spleen, which is negatively regulated by IL-22 [31]. Similarly, in *IFNAR*^−*/*−^ mice (interferon-α receptor deficiency) infected with ZIKV, γδ T cells were the predominant immune cells secreting IL-17A in splenic cells and brain tissues. The quantity of IL-17A-producing γδ T cells began to increase by day 3 post-infection and peaks by day 5 post-infection [32]. Compared to IFN-γ^+^ γδ T cells in peripheral blood, IL-17A^+^ γδ T cells predominantly accumulate in peripheral tissues, with their peak proliferation occurring at a later stage. As an inflammatory regulator, IL-17A activates various immune cells, thereby amplifying the immune response in the early stage. However, it is important to note that prolonged secretion of IL-17A may exacerbate tissue damage [33,34].

### The protective role of γδ T cells

γδ T cells provide protective immunity against flavivirus infections in mice, including enhanced survival rates, promotion of adaptive immunity, decreased viral loads, and amelioration of pathological injury to target organs. Firstly, γδ T cells enhance survival rates post-infection. In mouse models infected with lethal doses of WNV in wild-type (WT) mice and γδ T cell-deficient (*TCRδ*^*−/−*^) mice, it was observed that *TCRδ*^*−/−*^ mice experienced earlier and more rapid mortality compared to WT mice. When infected with sublethal doses of WNV, all *TCRδ*^*−/−*^ mice succumbed to infection, whereas 75% of WT mice survived [26]. Secondly, γδ T cells can play a role in the promotion of adaptive immunity. After 30 days post-initial infection, re-infection with a lethal dose of WNV in mice that survived initial infection revealed nearly complete survival in WT group, whereas mice in *TCRδ*^*−/−*^ group exhibited significantly reduced survival rates [35]. Thirdly, γδ T cells participate in the process of viral clearance. Compared to WT mice, *TCRδ*^*−/−*^ mice infected with WNV exhibited significantly higher viral loads in blood and brain tissues (target organs for WNV infection) on days 2 (early phase) and 6 (late phase) post-infection [26]. Similarly, in *TCRδ*^*−/−*^ mice infected with DENV, viral loads in the infection site (footpad) and DLNs were significantly higher than those in control groups [25]. Finally, γδ T cells may modulate the pathobiology of target tissues. In WNV-infected mice, brain tissues showed no inflammatory cells infiltration in WT mice on day 2 post-infection, whereas *TCRδ*^*−/−*^ mice exhibited mononuclear inflammatory cell infiltration, particularly in the ventricular walls near the basal ganglia. By day 6 post-infection, WT mice still showed minimal inflammatory cell infiltration in brain tissues, whereas *TCRδ*^*−/−*^ mice exhibited exacerbated inflammatory cell infiltration [26].

Additionally, an intriguing observation of two γδ T cell subsets (Vγ1^+^ T cells and Vγ4^+^ T cells) was made in WNV-infected mice of different ages: By day 3 post-infection, Vγ1^+^ T cells in splenic cells of young mice rapidly expanded and represented the major subset of γδ T cells producing IFN-γ, while Vγ4^+^ T cell expansion was less pronounced. Conversely, aged mice showed decreased proportions and proliferation rates of Vγ1^+^ T cells and higher proportions of Vγ4^+^ T cells compared to young mice [36]. Depletion of Vγ1^+^ T cells in young mice significantly increased viral loads in the brain and mortality rates, whereas depletion of Vγ4^+^ T cells resulted in significantly reduced viral loads in the brain and improved survival rates. Moreover, Vγ4^+^ T cells exhibited a greater ability to produce TNF-α, a cytokine known to contribute to blood-brain barrier compromise and facilitate WNV entry into the brain [36]. These findings indicate that Vγ1^+^ T cells play a protective role, whereas Vγ4^+^ T cells have the opposite effect.

## The protective mechanism of γδ T cells

As described above, γδ T cells play an important protective role in flavivirus infections. The protective mechanisms (**Table 2**) involved are as follows:

**Table 2:** The mechanisms of γδ T cells function in flavivirus infections. After flavivirus infection, γδ T cells can be activated through various recognition mechanisms of the γδ TCR and TLR signaling pathways. Subsequently, they secrete cytokines (IFN-γ, IL-17A, or TNF-α), enhancing the systemic immune response. However, we must be cautious, as the prolonged presence of certain cytokines may exacerbate tissue damage. Additionally, they can directly kill virus-infected cells.

The activation signaling	The secretion of cytokines	The direct killing effect
Recognition of phosphoantigens [41]	IFN-γ: (1) Inhibit the proliferation of infected cells [26]. (2) Promote the maturation of DCs and the formation of adaptive immunity [38].	γδ T cell-mediated cytotoxicity [41,42]
Mechanical contact of γδ TCR with EPCR [25]	IL-17A: (1) Promote the cytotoxic activity of CD8^+^ T cells in WNV [40]. (2) Serum levels of IL-17A are positively correlated with the severity of dengue fever patients [33].	
TLR7-MyD88 signaling pathway [37]	TNF-α: Vγ4^+^ T cells may enhance susceptibility of aged mice to WNV via increased secretion of TNF-α [36].	

EPCR, endothelial protein C receptor; DCs, dendritic cells; TLR7, toll-like receptor 7; MyD88, myeloid differentiation factor 88; IL17-RA, IL-17A receptor; DENV, dengue virus; WNV, West Nile virus; TNF-α, tumor necrosis factor α.

### The activation signaling

**Fig 1:**
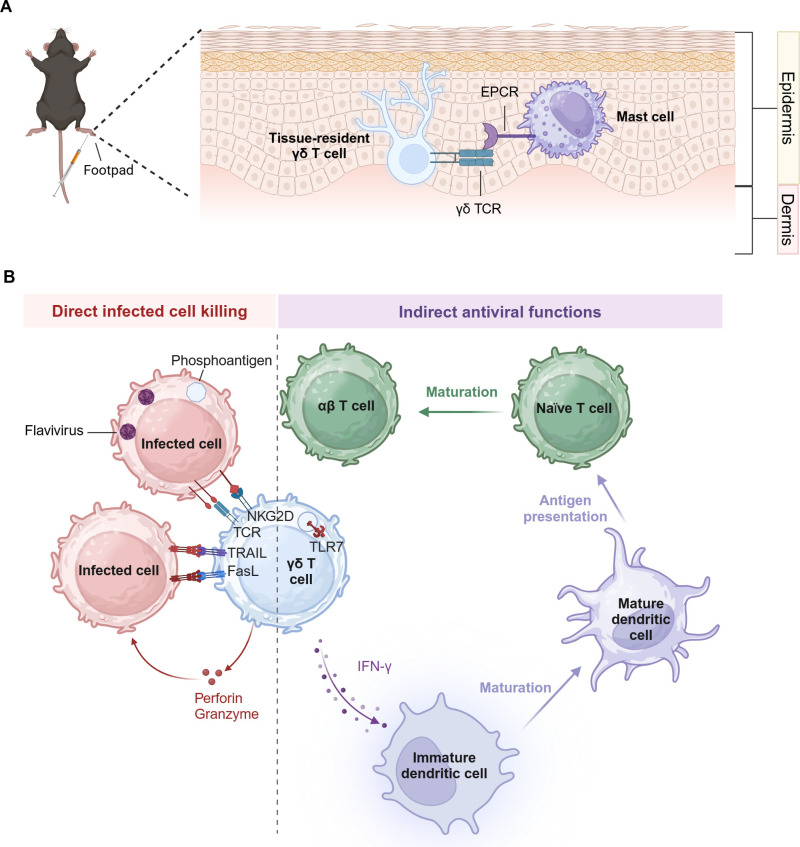
The protective mechanism of **γδ** **T cells in flavivirus infections.** **A. Mechanical contact between tissue-resident γδ TCR and endothelial protein C receptor (EPCR).** Following footpad injection of Dengue virus (DENV) in mice, skin-resident γδ T cells are the first to be activated. This activation occurs through mechanical interaction between their T cell receptor (TCR) and EPCR, which is upregulated on the surface of DENV-infected mast cells, thereby initiating their protective function. **B. Protective mechanism of γδ T cells in peripheral blood.** γδ T cell activation is primarily mediated through their TCR, which recognizes BTN proteins on the surface of infected cells. These BTN proteins undergo conformational changes in response to the intracellular accumulation of phosphoantigens, enabling TCR engagement. Furthermore, TLR7, a pattern recognition receptor that detects single-stranded RNA, has also been implicated in γδ T cell activation under certain conditions. Upon activation, γδ T cells execute their antiviral functions via two distinct mechanisms: direct and indirect pathways. The direct antiviral mechanism involves surface molecules such as NKG2D, TRAIL, and FasL, which detect the upregulation of corresponding ligands on infected cells. This interaction facilitates the targeted release of cytotoxic mediators, including perforin and granzyme B, resulting in the lytic destruction of infected cells. Conversely, the indirect antiviral mechanism is characterized by the secretion of IFN-γ, a cytokine that plays a pivotal role in facilitating the adaptive immune response, thereby augmenting viral clearance and immune defense. NKG2D, natural killer group 2 member D; TRAIL, tumor necrosis factor-related apoptosis-inducing ligand; FasL, Fas ligand; *Created in BioRender.*

γδ T cells can be activated through mechanical contact of their TCR with endothelial protein C receptor (EPCR) on mast cells during DENV infection. In Sash mice lacking mast cells, three days post-DENV infection, the number of γδ T cells at the infection site and in DLNs significantly decreased compared to the control group, accompanied by a marked reduction in IFN-γ secretion. This indicates a strong dependency of γδ T cell activation on mast cells [25]. Further experiments revealed that only mast cells exposed to DENV can activate γδ T cells, which subsequently kill DENV-infected dendritic cells (DCs), but spare mast cells. This suggests that mast cells may activate γδ T cells through a process akin to antigen presentation. Moreover, inhibition of TCR signaling in γδ T cells does not block their activation by mast cells, implying that mast cells require mechanical contact with γδ T cells to enhance their activation. Finally, it was observed that DENV infection increased surface expression of EPCR on mast cells; blocking EPCR with specific antibodies reduced the activation level and number of γδ T cells [25] (**Fig 1A**).

The TLR7-MyD88 signaling pathway has been demonstrated to participate in the activation of γδ T cells during WNV infection. *In vitro* treatment of mouse γδ T cells with TLR7 agonists showed increased activation, evidenced by significant upregulation of activation marker CD69 and elevated levels of Th1-type cytokines (IFN-γ and TNF-α) in the supernatant (**Fig 1B**). In *TLR7*^*−/−*^ and *MyD88*^*−/−*^ mice infected with WNV, significant reductions in the proportion of γδ T cells were observed on days 3 and 5 post-infection compared to controls [37].

### The secretion of cytokines

γδ T cells serve as the primary early source of IFN-γ during anti-WNV infection. Infection of *IFN-γ*^*−/−*^ mice with sublethal doses of WNV resulted in nearly all *IFN-γ*^*−/−*^ mice succumbing (90%), compared to 30% in the control group. Given that both αβ T cells and γδ T cells can produce IFN-γ, researchers further investigated the source of IFN-γ by assessing IFN-γ secretion among various cell subsets in splenocytes. By day 2 post-WNV infection in WT, *TCRβ*^*−/−*^, and *TCRδ*^*−/−*^ mice, significant levels of IFN-γ were detected in the blood and splenocytes of WT and *TCRβ*^*−/−*^ mice, whereas IFN-γ was nearly undetectable in *TCRδ*^*−/−*^ mice [26]. Additionally, the downregulation of IFN-γ secretion was observed in DCs from WNV-infected *TCRδ*^*−/−*^ mice compared to WT mice, evidenced by decreased surface activation markers CD40, CD80, CD86, and MHC-II. Moreover, co-culture experiments of DCs harvested from infected mice with CD4^+^ T cells showed significantly reduced activation levels of CD4^+^ T cells when DCs were derived from *TCRδ*^*−/−*^ mice [38] (**Fig 1B**).

γδ T cells may contribute to the regulation of adaptive immunity through the secretion of IL-17A, thereby exerting an indirect antiviral effect against flavivirus infections. As previously mentioned, *TCRδ*^*−/−*^ mice surviving initial WNV infection remained more susceptible to subsequent WNV infections compared to controls. Considering that γδ T cells may directly participate in anti-infection responses during secondary infections, researchers depleted γδ T cells in surviving WT mice prior to secondary infection. The results indicate that even in the absence of γδ T cells, nearly all mice survived, suggesting that γδ T cells do not exert a predominant anti-infection effect during secondary WNV infection [35]. Consequently, researchers evaluated the adaptive immunity of *TCRδ*^*−/−*^ and WT mice surviving initial WNV infection, finding no differences in IgM, IgG, and WNV-specific neutralizing antibody titers between the two groups. Subsequently, sera from both groups were transferred to naive mice prior to WNV challenge, and recipients from both groups successfully resisted lethal doses of WNV [35]. In a mouse model infected with JEV, *TCRδ*^*−/−*^ mice also exhibited comparable JEV-specific neutralizing antibody titers to WT mice [39]. These data suggest that γδ T cells do not impact the formation of flavivirus-specific neutralizing antibodies.

Therefore, researchers further analyzed the function of CD8^+^ T cells, demonstrating that naïve mice receiving splenocytes from surviving *TCRδ*^*−/−*^ mice had higher mortality rates compared to those receiving splenocytes from surviving WT mice, indicating a regulatory role of γδ T cells in the function of CD8^+^ T cells. Further functional analysis revealed that surviving *TCRδ*^*−/−*^ mice exhibited reduced numbers of CD8^+^ T cells post-infection compared to WT mice, along with diminished IFN-γ secretion and cytotoxic activity [35]. Another study showed that *IL-17A*^*−/−*^ mice infected with WNV displayed significantly reduced cytotoxic activity of CD8^+^ T cells and lower expression levels of cytotoxic genes compared to WT mice [40]. γδ T cells may promote the cytotoxic function of WNV-specific CD8^+^ T cells through IL-17A secretion, contributing to WNV clearance, but further research is warranted (**Fig 1B**).

### The direct killing effect

Upon activation, γδ T cells can directly lyse target cells infected with flaviviruses. Co-culturing human γδ T cells with WNV-infected cells resulted in significantly elevated levels of perforin in the supernatant and pronounced cytopathic effects compared to uninfected cells [41]. Similar observations were made in co-cultures of human γδ T cells with ZIKV-infected cells. Further investigation revealed that ZIKV infection upregulated expression of NKG2D ligands on target cells, and that blocking γδ T cell-mediated cytotoxicity against target cells can be achieved using NKG2D antibodies [42] (**Fig 1B**).

## Potential of γδ T cells-based anti-flaviviral therapies

Based on the aforementioned summary, it can be concluded that γδ T cells are the first responders during the early stage of flavivirus infections, providing antiviral defense while also facilitating the development of adaptive immunity. Therefore, in the context of limited treatment options for flavivirus infections, γδ T cells may offer a promising avenue for novel therapeutic strategies. Given the successful application of γδ T cell-based immunotherapies in the treatment of other diseases, this section discusses the possibility of applying the currently developed γδ T cells therapies to the treatment of flavivirus infections, hoping to give some suggestions for further research in this area (**Table 3**).

**Table 3:** γδ T cell-based anti-flaviviral potential therapies. Currently, there are five types of γδ T cell-based immunotherapies. These methods primarily utilize the specific recognition and activation mechanisms of γδ T cells to enhance their secretion of interferons and their cytotoxic ability against infected cells.

Strategies	Therapies	Mechanism	References
Expanded γδ T cells	IPP	Stimulate γδ T cells proliferation directly	[43]
	ZOL, PAM	Promote the accumulation of phosphoantigens	[8,41]
	ICT01	Promote the binding of BTN3A1 and BTN2A1	[44,46]
Bispecific antibody	Anti-γδ TCR and anti-flavivirus antigen	Promote the accumulation and activation of γδ T cells	[54]
	Flavivirus-reactive TCR and anti-CD3 scFV	Bring more general T cells to infected sites	[53]
CAR-γδ T cells	CAR-γδ T cells with flavivirus antigen	Kill infected cells more precisely	[61]
	CAR-γδ T cells with NKG2D	Induce more γδ T cells migration to infection sites	[63]
γδ TCR- T cells	αβ T cells with γδ TCR	Enhance T cells sensitivity to infected cells	[65]
	αβ T cells with γδ TCR, CD3 and flavivirus antigen-targeting Fab fragment	Promote activation and increase specificity of T cells	[64]
Drug-assisted γδ T cells	RO7020531	Enhance the cytotoxicity of γδ T cells via the activation of TLR7	[66,67]
	Anti-TIM-3 antibody	Promote the secretion of IFN-γ via the inhibition of TIM-3	[27,68,69]
	Vitamin C	Reduce apoptosis of γδ T cells	[70,71]
	AHCC	Promote the proliferation of γδ T cells and increase the production of IgM and IgG	[72]

IPP, isopentenyl pyrophosphate; ZOL, zoledronic acid; PAM, pamidronate; ICT01: agonist of BTN3A1; BTN, butyrophilin; scFV, single-chain variable fragment; CAR, chimeric antigen receptor; NKG2D, natural killer group 2 member D; RO7020531: agonist of TLR7; Tim-3, T cell immunoglobulin domain and mucin-domain-3; AHCC, active hexose correlated compound.

### Expanded γδ T cells

**Fig 2:**
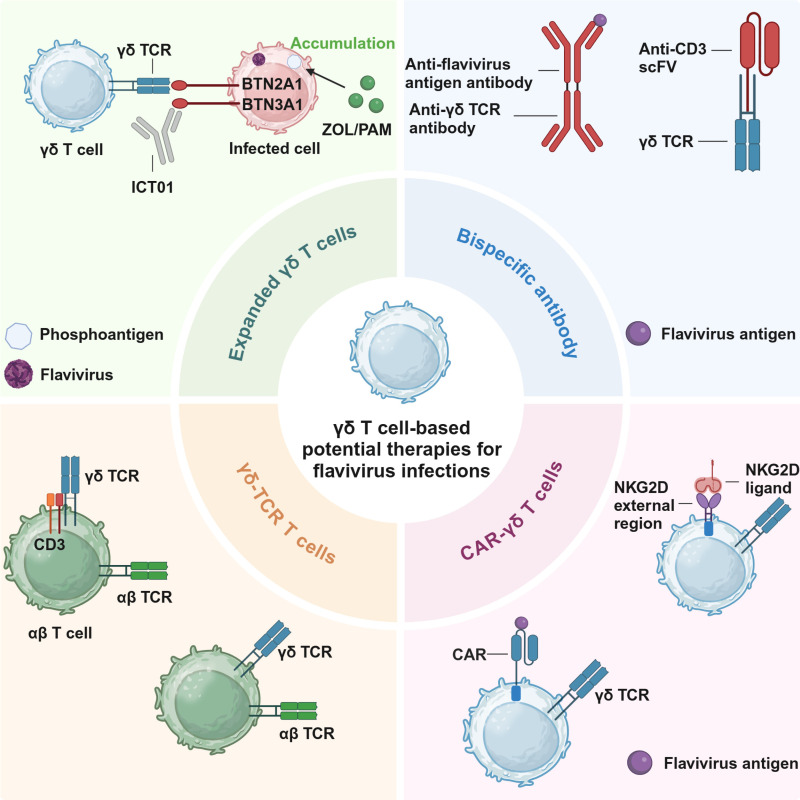
γδ T cell-based potential therapies for flavivirus infections. Current immunotherapies based on γδ T cells primarily include four main strategies. The first strategy involves the use of expanded γδ T cells, which is mainly based on the activation mechanism of γδ TCR. Zoledronic acid (ZOL) and pamidronate (PAM) can promote the accumulation of phosphoantigens within infected cells, which in turn induces conformational changes in BTN2A1 and BTN3A1, allowing them to interact with γδ TCR and activate γδ T cells. Additionally, the BTN3A1 activator ICT01 can directly induce conformational changes in BTN3A1, thereby activating γδ T cells through a phosphoantigen-independent pathway. The second strategy involves the use of bispecific antibodies, which are designed based on two approaches: the first approach aims to direct γδ T cells toward flavivirus-infected cells to enhance their specificity; the second utilizes the recognition properties of γδ TCR to target αβ T cells to the site of infected cells. The third strategy builds upon γδ T cells by introducing artificially engineered chimeric antigen receptors (CARs) designed to target flavivirus antigens or NKG2D ligands specifically upregulated in infected cells, thereby further enhancing the cytotoxic function of γδ T cells. The fourth strategy leverages the unique recognition characteristics of γδ TCR to modify αβ T cells, endowing them with similar recognition capabilities to γδ T cells, thereby increasing their ability to kill infected cells. NKG2D, natural killer group 2 member D. *Created in BioRender.*

Based on the mechanism of γδ T cell activation by pAgs, the administration of human PBMCs with pAgs leads to rapid and selective expansion of Vγ9Vδ2 T cells *in vitro* [43]. Furthermore, amino-bisphosphonates (such as Zoledronic acid and pamidronate), by inhibiting farnesyl pyrophosphate synthase and disrupting the mevalonate pathway, result in intracellular accumulation of pAgs, thereby specifically activating Vγ9Vδ2 T cells [8,41]. Additionally, the BTN3A1 monoclonal antibody 20.1 directly binds BTN3A1, inducing a conformational change that facilitates binding with BTN2A1, thus stimulating proliferation of Vγ9Vδ2 T cells in an pAgs-independent manner [44]. Furthermore, in humanized mice infected with influenza A virus, treatment with amino-bisphosphonates improved several protective indicators, including body weight, lung tissue pathology, viral load, and mortality [8]. Of note, amino-bisphosphonates have already been clinically applied in the treatment of Paget’s disease [45]. Additionally, the BTN agonist ICT01 has also demonstrated promising clinical efficacy in cancer therapy and is considered safe for human use [46] (**Fig 2**). Excitingly, due to their HLA-independent, allogeneic Vγ9Vδ2 T-cell therapy—where Vγ9Vδ2 T cells from healthy donors are expanded and infused into patients—has been shown to prolong the survival of cancer patients without causing adverse effects [47]. In the future, patients with acute flavivirus infections may potentially receive pre-expanded Vγ9Vδ2 T cells from healthy donors to control the progress of acute viral infection. Therefore, the therapeutic potential of Vγ9Vδ2 T cells could be rapidly evaluated in mouse models and subsequently advanced to clinical trials for the treatment of flavivirus infections.

In addition to Vγ9Vδ2 T cells, the challenge of expanding Vδ1 T cells has been successfully addressed, resulting in the development of delta one T (DOT) cells suitable for clinical use [48]. Preclinical studies have demonstrated that DOT cells can effectively suppress cancer progression in xenograft models [49]. Importantly, DOT cells produce abundant IFN-γ and TNF-α but do not secrete IL-17A [48]. Notably, IL-17A has been shown to play a protective role in WNV infections but to exacerbate disease severity in DENV infections [33,40]. Therefore, the safety and efficacy of DOT cells need to be further evaluated across different flaviviruses. Overall, given the absence of human clinical trials and the limited research related to flaviviruses, DOT cells should not currently be considered a primary therapeutic option for the treatment of flavivirus infections.

### Bispecific antibody

In HCMV [50], hepatitis B virus (HBV) [51], and HIV [52] infections, there has been preliminary research on T cell-engaging bispecific antibodies (bsAbs). One arm of bispecific antibodies targets viral-specific antigens, while the other arm targets antibody molecules on T cell surfaces (such as CD3, Vγ9), thereby directing T cells towards infected cells. However, the efficacy of CD3-targeting bsAbs may be limited since they activate T cells across all lineages [53]. Recruiting γδ T cells with defined protective roles (such as Vγ9Vδ2 T cells) to kill infected cells may offer advantages over CD3-based bsAbs. Moreover, Vγ9Vδ2 T cells combined with bsAbs can still be activated by pAgs, further enhancing their cytotoxic functions [54]. Additionally, Vγ9-based bsAbs have been tested for phase I clinical trial [54]. Therefore, one arm of the bsAbs designed for the treatment of flavivirus infections could be targeted against the E protein or NS1 protein (**Fig 2**). The E protein, as a structural component of flaviviruses, is a critical epitope for neutralizing antibodies [55]. Although the NS1 protein, a non-structural component of flaviviruses, is not expressed on the viral surface, it is secreted into the bloodstream during the early stage of infection and is thus commonly used as an early biomarker for flavivirus infections [56]. Furthermore, the antibody structures targeting these two proteins have already been extensively characterized [57,58], significantly reducing the complexity of bsAbs design. Therefore, this therapeutic approach should be given priority consideration.

### CAR-γδ T cells

Chimeric antigen receptor (CAR)-T cell therapy has been widely employed in cancer treatment, primarily by genetically modifying αβ T cells to introduce receptor genes capable of recognizing tumor-specific antigens, thereby achieving precise targeted killing. However, as a personalized therapy, CAR-T requires extensive preparation and incurs high costs. Allogeneic transplantation may lead to severe graft-versus-host disease (GVHD) and cytokine release syndrome [59]. In contrast to αβ T cells, both NK cells and γδ T cells do not require prior sensitization and do not cause GVHD, making them increasingly attractive in CAR-T cell therapy. CAR-NK cells targeting the S protein of SARS-CoV-2 enhanced the production of TNF-α and IFN-γ upon binding to viral particles, thereby augmenting cytotoxicity against cells expressing high levels of the spike protein [60]. However, CAR-NK cell therapy faces challenges such as difficulties *in vivo* expansion and low transfection efficiency. Conversely, CAR-γδ T cell therapy based on Vγ9Vδ2 T cells can overcome the limitations of CAR-T and CAR-NK cells, promising significant application prospects [61]. Furthermore, expanded CAR-Vγ9Vδ2 T cells retain cross-presenting capabilities, enabling direct antigen presentation to αβ T cells and enhancing overall immune response against infections [62]. Elevated expression of NKG2D ligands were observed in target cells infected with ZIKV [42]. Therefore, in addition to targeting viral antigens, CAR-γδ T cells can be engineered to target NKG2D ligands, further enhancing their targeted killing function [63]. In conclusion, the engineering of Vγ9Vδ2 T cells derived from healthy donors, incorporating flaviviruses antigens or NKG2D-CAR, could significantly enhance the targeting capability of Vγ9Vδ2 T cells (**Fig 2**). However, such approaches should be pursued primarily in scenarios where allogeneic Vγ9Vδ2 T cells therapy fails to achieve sufficient therapeutic efficacy and requires further optimization.

### γδ TCR-T cells

Traditional TCR-T cell therapy involves screening and identifying αβ TCR sequences that specifically bind target antigens. Through genetic engineering, these sequences are transduced into the nuclei of patient’s conventional T cells, enabling them to express αβ TCR sequences for specific recognition and killing of target cells. In HBV, infusion of HBV-specific CD8^+^ Vβ^+^ T cells into HBV-related hepatocellular carcinoma patients resulted in decreased or stable levels of HBsAg and HBV DNA in most patients’ circulation, highlighting the therapy’s significant targeting efficacy [64]. Leveraging the rapid target cell recognition capability of γδ TCR involves transducing γδ TCR into conventional T cells to enhance their sensitivity to target cells. Furthermore, fusion of γδ TCR with T cell stimulatory molecule CD3 and viral antigens further enhances signal transduction and targeting of γδ TCR-T cells [65] (**Fig 2**). Since this approach does not directly utilize Vγ9Vδ2 T cells but leverages the recognition mechanism of the Vγ9Vδ2 TCR to enhance the specificity of αβ T cells, it necessitates consideration of MHC restriction, which may lead to the occurrence of GVHD. Therefore, this approach should not currently be considered for the treatment of flavivirus infections.

### Drug-Assisted γδ T cells

TLR7, as a crucial pattern recognition receptor for flaviviruses, is involved in the activation of γδ T cells during WNV infection [37]. TLR7 agonists serve as adjuvants in cancer immunotherapy clinical trials. Pre-treatment of tumor cells with TLR7 ligands significantly enhances the cytotoxicity of γδ T cells [66]. A current phase I clinical study demonstrated that the TLR7 agonist RO7020531 was effective and well-tolerated in patients with chronic hepatitis B [67]. Therefore, TLR7 agonists may hold promise for treating flavivirus infections.

TIM-3, a negative regulator of immune cells, suppresses the Th1 cytokine secretion and cytotoxicity of Vγ9Vδ2 T cells when elevated. Blocking TIM-3 enhances the function of Vγ9Vδ2 T cells [68,69]. Increased expression of the TIM-3 molecule on IFN-γ^+^ γδ T cells was observed in patients with acute dengue fever [27], suggesting that TIM-3 upregulation may impair the anti-infective function of γδ T cells, potentially contributing to the manifestation of clinical symptoms. Therefore, anti-TIM-3 therapy may benefit patients infected with DENV.

Vitamin C (VC) is an essential vitamin that plays a crucial role in regulating immune cell functions. A research study indicated that VC reduces apoptosis of γδ T cells and increases secretion of Th1 cytokines [70]. In patients infected with ZIKV, high-dose intravenous VC administration alleviated clinical symptoms without adverse effects [71]. However, further clarification is needed regarding the role of γδ T cells in this context.

In a mouse model of WNV infection, oral administration of active hexose correlated compound (AHCC) one week before infection, on day 1 and 3 post-infection enhanced mice resistance against lethal doses of WNV. Moreover, an increase in γδ T cell numbers and specific IgM and IgG production against WNV was observed [72]. These findings suggest that dietary supplementation with AHCC may enhance γδ T cell function, thereby prophylactically boosting host resistance against flaviviruses (**Table 3**).

### Potential role in vaccine

In addition to their direct anti-infective role, γδ T cells function as part of adaptive immunity, undergoing significant expansion and generating memory-like responses during secondary viral infections [6,73], and promoting the formation of adaptive immunity through antigen presentation and cytokine secretion [38,74]. In rhesus macaques, following immunization with YF-17D (a live attenuated YFV vaccine) and recombinant adenovirus type 5 vaccines, an increase in IFN-γ was observed only in the YF-17D group. Further data analysis revealed an early increase in the proportion of IFN-γ^+^ γδ T cells, highlighting the significant role of γδ T cells in vaccine immunity [75]. Moreover, studies indicated that short-term oral administration of γδ T cell modulator AHCC in subjects vaccinated against influenza virus enhanced titers of influenza-specific antibodies and increased the number of CD8^+^ T cells [76]. Additionally, a recent study has demonstrated that the measles, mumps, and rubella vaccine can induce γδ T cells to develop trained immunity, a phenomenon in which innate immune cells exhibit an enhanced functional response upon re-exposure to pathogens or unrelated stimuli [77]. This effectively leverages the non-MHC-restricted characteristics of γδ T cells, thereby broadening the protective spectrum of the vaccine. Consequently, future flavivirus vaccine designs should consider incorporating strategies to activate γδ T cells to enhance their efficacy.

In summary, current γδ T cell-based immunotherapies predominantly focus on Vγ9Vδ2 T cells due to their well-defined mechanisms of specific activation and expansion, established manufacturing processes, and favorable clinical safety profiles. Therefore, future development of γδ T cell immunotherapies for flavivirus infections should prioritize this subset. Among the various methods discussed, amino-bisphosphonates, which are already in clinical use, and the clinically safe BTN agonist ICT01 should be considered as primary candidates for therapeutic trials targeting flavivirus infections. Additionally, immunomodulators such as VC and AHCC could serve as complementary approaches. Furthermore, the design of bispecific antibodies (bsAbs) should integrate flavivirus-specific antigens, such as the E protein or NS1 protein, with Vγ9-targeting antibodies. Moreover, flavivirus vaccine development should explore strategies to specifically activate γδ T cells. These two approaches, when rationally designed, could be evaluated in preclinical animal models to assess their efficacy and translational potential.

## Conclusion marks

Overall, the available evidence indicates that γδ T cells play a protective role in flavivirus infections. The immunotherapies based on γδ T cells can be considered as a potential therapeutic approach for flavivirus infections. However, several key questions remain to be further elucidated: (i) the immune characteristics and roles of various γδ T cell subsets (Vδ1, Vδ2, Vδ3) in flavivirus-infected patients. Currently, our focus has been predominantly on the Vδ2 subset during flavivirus infections. However, the unique recognition patterns of the Vδ1 subset in viral infections and the significant increase in the proportion of Vδ1^−^ Vδ2^−^ cell subsets in ZIKV-infected patients necessitate investigation into the roles of other γδ T cell substructures in flavivirus infections. (ii) The protective mechanism of γδ T cells needs to be further explored. For example, γδ T cells not only process endogenous antigens to induce activation of CD4^+^ αβ T cells but also uptake and cross-present soluble antigens to activate CD8^+^ αβ T cells in HCMV infections [6]. If we can clarify whether γδ T cells play this role in flavivirus infections, it may be meaningful for vaccine design and immunotherapy strategies. (iii) The efficacy and safety of γδ T cell-based therapies in flavivirus infections. Currently, effective treatments for flaviviruses are lacking. Exploring the safety and efficacy of these therapies in flavivirus infections could alleviate the disease burden. Based on current treatment strategies, we provide a framework for prioritizing therapeutic approaches. Addressing these questions will improve our understanding of the role γδ T cells play in flavivirus infections and promote the safe application of γδ T cell-based therapeutic strategies in their treatment.

## Key papers

- Pierson TC, Diamond MS. The continued threat of emerging flaviviruses. Nat Microbiol. 2020;5(6):796-812. https://doi.org/10.1038/s41564-020-0714-0 PMID: 32367055 [3].
- Caron J, Ridgley LA, Bodman-Smith M. How to Train Your Dragon: Harnessing Gamma Delta T Cells Antiviral Functions and Trained Immunity in a Pandemic Era. Front Immunol. 2021;12:666983. https://doi.org/10.3389/fimmu.2021.666983 PMID: 33854516 [7].
- Mantri CK, St John AL. Immune synapses between mast cells and γδ T cells limit viral infection. J Clin Invest. 2019;129(3):1094-108. https://doi.org/10.1172/JCI122530 PMID: 30561384 [25].
- Cimini E, Grassi G, Beccacece A, Casetti R, Castilletti C, Capobianchi MR, et al. In Acute Dengue Infection, High TIM-3 Expression May Contribute to the Impairment of IFNγ Production by Circulating Vδ2 T Cells. Viruses. 2022;14(1). https://doi.org/10.3390/v14010130 PMID: 35062334 [27].
- Wang T, Gao Y, Scully E, Davis CT, Anderson JF, Welte T, et al. Gamma delta T cells facilitate adaptive immunity against West Nile virus infection in mice. J Immunol. 2006;177(3):1825-32. https://doi.org/10.4049/jimmunol.177.3.1825 PMID: 16849493 [35].

## Learning points

- Due to the acute onset of flavivirus infections and the lack of rapid adaptive immune responses, the innate immune system, particularly γδ T cells, plays a helpful role in early defense mechanisms.
- γδ T cells are a major component of the innate immune system and can rapidly respond to infections by exhibiting cytotoxic activity and secreting antiviral cytokines. During flavivirus infections, their numbers and functions undergo changes.
- In the context of the lack of flavivirus treatment, the potential application of γδ T cells-based therapies could be considered into flavivirus treatment, hoping to give some suggestions for the exploration of new therapeutic strategies.
